# The burden of anxiety among people living with HIV during the COVID-19 pandemic in Pune, India

**DOI:** 10.1186/s12889-020-09656-8

**Published:** 2020-10-23

**Authors:** Ivan Marbaniang, Shashikala Sangle, Smita Nimkar, Kanta Zarekar, Sonali Salvi, Amol Chavan, Amita Gupta, Nishi Suryavanshi, Vidya Mave

**Affiliations:** 1 Byramjee Jeejeebhoy Government Medical College - Johns Hopkins University Clinical Research Site, Jai Prakash Narayan Road, Maharashtra 411001 Pune, India; 2 Department of Epidemiology, McGill University, Purvis Hall, 1020 Pine Avenue, Montreal, QC H3G 1A2 Canada; 3 Department of General Medicine, Byramjee Jeejeebhoy Government Medical College, Pune, Maharashtra India; 4 Department of Medicine and Infectious Diseases, Johns Hopkins University, Baltimore, MD USA

**Keywords:** India, COVID-19 pandemic, Poverty, GAD-7, Anxiety, Screening

## Abstract

**Introduction:**

Globally, India has the third largest population of people living with HIV (PLHIV) and the second highest number of COVID-19 cases. Anxiety is associated with antiretroviral therapy (ART) nonadherence. It is crucial to understand the burden of anxiety and its sources among Asian Indian PLHIV during the COVID pandemic, but data are limited.

**Methods:**

During the first month of government mandated lockdown, we administered an anxiety assessment via telephone among PLHIV registered for care at a publicly funded antiretroviral therapy (ART) center in Pune, India. Generalized anxiety was defined as GAD-7 score ≥ 10. Sociodemographic and clinical variables were compared by anxiety status (GAD-7 score ≥ 10 vs GAD-7 score < 10). Qualitative responses to an open-ended question about causes of concern were evaluated using thematic analysis.

**Results:**

Among 167 PLHIV, median age was 44 years (IQR 40–50); the majority were cisgender women (60%) and had a monthly family income < 200 USD (81%). Prior history of tuberculosis and other comorbidities were observed in 38 and 27%, respectively. Overall, prevalence of generalized anxiety was 25% (*n* = 41). PLHIV with GAD-7 score ≥ 10 had fewer remaining doses of ART than those with lower GAD-7 scores (*p* = 0.05). Thematic analysis indicated that concerns were both health related and unrelated, and stated temporally. Present concerns were often also projected as future concerns.

**Conclusions:**

The burden of anxiety was high during COVID lockdown in our population of socioeconomically disadvantaged PLHIV in Pune and appeared to be influenced by concerns about ART availability. The burden of anxiety among PLHIV will likely increase with the worsening pandemic in India, as sources of anxiety are expected to persist. We recommend the regular use of short screening tools for anxiety to monitor and triage patients as an extension of current HIV services.

## Introduction

The global COVID-19 pandemic and the subsequent lockdowns authorized by governments as containment measures have had a profound impact on mental health. Studies from diverse settings have consistently reported an increase in the burden of mental health conditions during this period [1–3]. The differential power structures that shape social hierarchies are likely instrumental in making certain groups, such as people living with HIV (PLHIV), at higher risk of developing more serious mental health issues [4]. PLHIV already have a disproportionately higher burden of mental health conditions [5], yet the impact of the pandemic is presently not well-understood in this potentially vulnerable population.

From a public health standpoint, it is important to understand the relationship between mental health among PLHIV, HIV treatment outcomes and HIV transmission dynamics. Depression is associated with treatment failure [6, 7], lower CD4 counts and risky sexual behavior [8, 9], and anxiety is associated with disengagement from care [10, 11]. The importance of generating mental health data among PLHIV in the setting of the pandemic can be inferred given that worsening mental health has been reported in the general population.

Globally, India has the third largest population of PLHIV [12] and had the second highest number of confirmed COVID-19 cases as of September 24, 2020 [13]. A recent meta-analysis of studies from India identified anxiety or depression as risk factors for nonadherence to antiretroviral therapy (ART) [14]. Another meta-analysis that included data from India found that anxiety was associated with 70% higher odds of ART nonadherence [15]. India’s HIV response already functions within an overburdened system [16]. Declining mental health during the pandemic could have a crippling effect on a healthcare system that has been further paralyzed by the rapidly increasing number of COVID-19 cases [17] following the easement of lockdown restrictions on June 8, 2020 [18].

Taking into consideration the findings of the two aforementioned recent meta-analyses; assuming that in a crisis like the COVID-19 pandemic, anxiety precedes depression; and recognizing that the two conditions frequently coexist [19], we sought to quantify the prevalence of anxiety symptoms, and the sources of anxiety among PLHIV in Pune, India.

## Methods

### Study population and procedures

Between April 21, 2020 and May 28, 2020, we recruited a subset of adults (≥18 years) enrolled in an ongoing 48-month prospective cohort study who needed to re-schedule their 4-year study visit due to the government mandated lockdown. The parent study seeks to understand the development of non-communicable diseases among PLHIV; the details have been reported elsewhere [20]. Briefly, participants (*n* = 400) were registered for care at the ART center affiliated with Byramjee Jeejeebhoy Government Medical College and Sassoon General Hospitals (BJGMC–SGH), a publicly-funded tertiary healthcare center in Pune, Maharashtra. The ART center functions under India’s National AIDS Control Organization (NACO) and currently provides care to approximately 5000 PLHIV from lower and lower-middle socioeconomic backgrounds. Pune has consistently reported high HIV prevalence compared to the national average (0.67% versus 0.31%) [21, 22], and Maharashtra is the state most affected by the pandemic, contributing approximately one third of all confirmed COVID-19 cases in India [23].

### Ethics

Two parent study counselors contacted potential participants via telephone (*n* = 323) to reschedule their study visit and also obtained verbal consent for the present study in Marathi, the locally spoken language. Participants had previously consented to being contacted on the phone number used. The Ethics Committee of BJGMC-SGH and Johns Hopkins University approved this project.

### Procedures

Sociodemographic and clinical data were obtained from the parent study. Updated information on marital status, employment, income, CD4 counts and viral loads were obtained from contacted participants. All consenting participants completed: 1) a single General Anxiety Disorder–7 (GAD-7) assessment (available online free of charge) [24], which uses a series of 7 questions to assess anxiety symptoms over the previous 2 weeks; 2) a COVID-19 symptom screen based on the US Centers for Disease Control (CDC) checklist [25]; 3) and history of exposure to COVID-19; and 4) an open-ended question, which was added after the first 38 participants were recruited, “In the present situation, what is/are the most important thing(s) that you are worried about?” Responses to the open-ended question were recorded and translated verbatim from Marathi into English by a translator proficient in both languages.

### Outcomes

Primary outcomes were generalized anxiety disorder at a single timepoint and causes for concern, as reported in response to the open-ended question. Generalized anxiety disorder was defined as GAD-7 score ≥ 10, which has been shown to have 89% sensitivity and 82% specificity [26, 27]; no generalized anxiety disorder was defined as GAD-7 score < 10.

### Statistical and qualitative analysis

Sociodemographic and clinical characteristics were compared among participants with and without generalized anxiety disorder (GAD score ≥ 10 vs. GAD score < 10). Wilcoxon rank sum and Fisher’s exact tests were used to evaluate differences in continuous and categorical variables, respectively. As sensitivity analyses, additional GAD-7 score cutoffs (5, 8 and 15) were used to define generalized anxiety disorder. A two-tailed *p*-value of 0.05 was used to infer statistical significance. All analyses were performed using Stata version 16.0.

Qualitative responses to the open-ended question were examined using thematic analysis. Two authors (IM and SN) independently coded these data using an inductive approach. Codes and themes were identified directly from the responses. The results we present are situated within a broadly essentialist framework where the material and experiential reality of participants was taken at face value [28]. Codes and themes were organized in NVivo 12.

## Results

### Study population

Of 323 potential participants, 167 (52%) were able to be contacted, and all were included in the quantitative analysis. Among those not able to be contacted after three attempts on two separate days (*n* = 156), 36% chose not to receive phone calls, 33% were out of cellular coverage area, and 25% had provided a phone number that was no longer in use. Overall (*n* = 167), median age was 44 years (IQR 40–50), 60% were cisgender women (*n* = 100), 81% had a monthly household income below 200 USD, and 57% had been employed in the informal sector prior to the lockdown. A significant proportion were not aware of their latest CD4 counts (40%) or viral loads (43%), 38% had a history of tuberculosis, 27% (*n* = 45) were living with another comorbid illness, and two participants reported exposure to symptomatic SARS-CoV-2 individuals, but none of the participants reported positive symptomatology (Table 1).

**Table 1:** Distribution of GAD-7 scores of by sociodemographic and clinical characteristics among PLHIV contacted

	Total N (%)	GAD-7 ≥ 10 n (%)	GAD-7 < 10 n (%)	*p*-value
N (%)	**167**	**41 (24.6)**	**126 (75.4)**	–
Median age in years (IQR)	44 (40–50)	43 (40–49)	45 (40–50)	0.8
Gender				
Cisgender men	66 (39.5)	17 (41.5)	49 (38.9)	0.3
Cisgender women	100 (59.9)	23 (56.1)	77 (61.1)	
Transgender woman	1 (0.6)	1 (2.4)	0	
Monthly household income (USD)				
< 65	35 (20.9)	11 (26.8)	24 (19.0)	0.6
65–130	64 (38.3)	14 (34.1)	50 (39.6)	
131–199	36 (21.6)	10 (24.4)	26 (20.6)	
≥ 200	32 (19.2)	6 (14.6)	26 (20.6)	
Education				
No formal education	22 (13.2)	4 (9.8)	18 (14.3)	0.4
≤ 9 years	74 (44.3)	22 (53.7)	52 (41.3)	
> 9 years	71 (42.5)	15 (36.6)	56 (44.4)	
Employment prior to lockdown ^a^				
Unemployed	33 (19.8)	9 (21.9)	24 (19.0)	0.5
Informal sector	95 (56.9)	25 (61.0)	70 (55.6)	
Salaried	39 (23.3)	7 (17.1)	32 (25.4)	
Living with a spouse				
Yes	83 (49.7)	19 (46.3)	64 (50.8)	0.7
No	84 (50.3)	22 (53.7)	62 (49.2)	
Median duration on ART in years (IQR)	9.8 (6.5–12.9)	9.5 (6.7–11.8)	9.9 (6.4–13.1)	0.4
Latest CD4 counts (cells/mm^3^)				
< 500	44 (26.4)	10 (24.4)	34 (26.9)	0.8
≥ 500	57 (34.1)	13 (31.7)	44 (34.9)	
Do not know	66 (39.5)	18 (43.9)	48 (38.1)	
Latest viral load				
Undetectable (< 50 copies/mL)	87 (52.1)	16 (39.0)	71 (56.3)	0.1
≥ 50 copies/mL	9 (5.4)	3 (7.3)	6 (4.8)	
Do not know	71 (42.5)	22 (53.7)	49 (38.9)	
Prior history of tuberculosis				
Yes	64 (38.3)	19 (46.3)	45 (35.7)	0.3
No	103 (61.7)	22 (53.7)	81 (64.3)	
Living with another comorbidity ^b^				
Yes	45 (27.0)	12 (29.3)	33 (26.2)	0.7
No	122 (73.0)	29 (70.7)	93 (73.8)	
Median days of remaining ART (IQR)	60 (28–76)	32 (17–60)	60 (30–79)	**0.05**
Discontinued ART during the lockdown	5 (3.3)	–	–	–

*ART* Antiretroviral Therapy

Median GAD-7 score for the study population was 3 (IQR: 0–9), range 0–21

^a^ Informal sector employment for women mainly included working as house maids or domestic help (89%), for men this was mainly as daily wage laborers (92%)

^b^ Comorbidity includes having any of the following: COPD, asthma, CVD, hypertension, diabetes, renal disease, cancer

Thematic analysis was conducted on a subset of 122 participant responses, after excluding the first 38 participants (for whom the open-ended question had not been asked) and an additional 7 participants who declined to answer the question.

### Prevalence of anxiety

The GAD-7 scale had high internal consistency among our study population (Cronbach’s alpha 0.96). Overall, approximately 25% of participants (*n* = 41/167) reported anxiety symptoms consistent with generalized anxiety disorder. Participants with GAD-7 scores ≥10 were more likely to have fewer remaining days of ART medications (*p* = 0.05) compared to those with scores below 10, and this difference remained significant when the GAD-7 cutoff was raised to 15 (*p* = 0.02). Other variables did not differ significantly by GAD-7 score, even when alternative cutoffs were applied. When stratified by gender, cisgender men with GAD-7 scores ≥10 were more likely to be living without a spouse and marginally more likely to have a monthly family income below 130 USD (*p* = 0.09) than those with scores below 10; among cisgender women, GAD-7 scores were independent of living with a spouse (supplementary file). These gender-specific findings were no longer significant when the GAD-7 cut-off was changed to 5, 8 or 15, indicating that significance was a function of the cut-off used and hence unreliable.

### Causes of anxiety

The following four themes were identified in thematic analysis: a) concerns related to the immediate present; b) concerns related to the imminent future; c) lack of social and financial support; and d) indifference to circumstances secondary to COVID-19; themes a) and b) were further classified as health-related or health-unrelated. Cognizant of the qualitative framework of thematic analysis, we do not quantify the exact number of participants who expressed each theme. However, two-thirds expressed themes A and B, roughly one half expressed theme C, and one third expressed theme D.

#### Theme a: concerns associated with the immediate present

Immediate health-related concerns were articulated as perceived increased susceptibility to COVID-19 or beliefs of being infected with COVID-19 in the absence of symptoms. These appeared to directly stem from participants’ self-awareness of immunodeficiency due to HIV.

“I have low CD4 counts and I am also taking medicines for tuberculosis. I am scared that I will get infected with coronavirus.” (cisgender man, GAD-7 score: 2)

“I have low immunity because of HIV, I am worried of getting COVID-19 infection. I feel that even a common cold could be coronavirus.” (cisgender woman, GAD-7 score: 10)

Immediate non health-related concerns centered around financial insecurity resulting from unemployment and a lack of savings and predominantly drove apprehensions about food security, eviction, and the ability to provide for the family.

“I am a construction worker. I am at home with my two children. My wife is dead. Currently I am worried about how the house will run as there is no money and no work.” (cisgender man, GAD-7 score:12)

“As the only earning member of my family, I am worried. My children are young. We are doing whatever it takes to get by, but because of the lockdown I am unemployed now. The house is rented. I cannot return to my village either.” (cisgender man, GAD-7 score: 6)

“There is no food at home currently and I cannot feed my children. I am a housewife and I have no income or savings. The children used to earn by washing cars.” (cisgender woman, GAD-7 score: 10)

#### Theme B: concerns associated with the imminent future

Imminent health-related concerns were articulated as apprehension about COVID-19 persistence continuing to endanger personal health following a reopening.

“I work as a care counsellor in the ART center. There are no coronavirus patients at this time point, but I am worried what will happen if they visit the center in the future?” (cisgender man, GAD-7 score: 1)

“I am scared to return to get my medicines at the ART center after the lockdown, if coronavirus does not end. Coronavirus must end.” (cisgender woman, GAD-7 score: 5)

Imminent non health-related concerns included fears about **s**hortages of opportunities for gainful employment or dismissal from current employment and fed into anxieties about an uncertain future that such eventualities would ensue. Such fears often co-existed with an anticipation for “normality”.

“I am a sex worker. My business is closed and I have no clients because of the lockdown. I will die of hunger if the virus continues. I am worried all the time. If coronavirus doesn't end, then what?” (cisgender woman, GAD-7 score: 5)

“I am going to lose my job because of this lockdown. I am eager to know when will COVID-19 end, when will we go back to normal life?” (cisgender woman, GAD-7 score: 9)

“I stay with my mother and sold fruits for a living. Now that has closed, and I don’t know when I will be able to start again. When will COVID-19 end? When can we start normal life?” (cisgender woman, GAD-7 score: 12)

#### Theme C: lack of social and financial support

Isolation from family members and friends accompanied feelings of loneliness and helplessness, and the lack of financial buffers perpetuated these feelings.

“I stay alone. I used to run a beauty salon that I rented, which is now closed. I have no money to pay the owner who is asking for rent. I have no savings and no one to talk to. I have a lot of tension and I feel lonely.” (cisgender woman, GAD-7 score: 21)

“I stay alone. My daughter is recently married. I worked in a company, but it has closed. I have no salary and I stay in a rented house. I receive no help from my in-laws who stay in the same neighborhood.” (cisgender woman, GAD-7 score: 10)

This theme was also common among migrant workers from outside or within the state.

“My family is in Bihar (a state 900 miles to the east). I want to go home, but I can't. There is a lot of tension and I worry a lot. I have no work and no money now.” (cisgender male, GAD-7 score: 21)

#### Theme D: indifference to circumstances secondary to COVID-19

Some remained unperturbed by the pandemic and its control measures. However, this indifference appeared to be closely linked to a sense of security by virtue of a profession, continuing employment or location.

“I work in the fields. There is no coronavirus there. Everything is fine.” (cisgender man, GAD-7 score: 0)

“Now, I have work on the sewing machine, and I am not worried at all.” (cisgender woman, GAD-7 score: 0)

“I do not get out of the house and I am not worried at all.” (cisgender woman, GAD-7 score: 0)

## Discussion

This survey found high prevalence of generalized anxiety disorder (GAD-7 score ≥ 10) among PLHIV during the lockdown due to COVID-19 in Pune, India. More severe anxiety was not differentially distributed by age, gender, or socioeconomic background, underscoring the pervasiveness of anxiety symptoms in the current pandemic. Additionally, a range of health-related and health unrelated factors directly linked to the pandemic, affected participants’ perceptions, and shaped their present beliefs and future expectations. Anxiety symptoms pervasive in the current pandemic, have implications for HIV outcomes.

One study from Hong Kong conducted during the early phases of the pandemic used the GAD-7 scale and found marginally lower prevalence of anxiety among HIV uninfected individuals with age and gender distributions comparable to our study population [29]. However, the largest study to report on anxiety symptoms among Asian Indian PLHIV found notably higher prevalence than our study [30]. We primarily attribute this incongruency to different scales and classifications used among these studies, but acknowledge that perceptions of diminished vulnerability to COVID-19 among some individuals, as evidenced in our qualitative findings, could also play a role. It is, therefore, imperative that our results not be interpreted in isolation, but rather in the context of the evolving pandemic in India.

As mentioned earlier, nonadherence to ART is one of many adverse effects of anxiety [14, 15]. While our study population is appreciably small, potential nonadherence among 25% could have far reaching consequences for viral suppression, HIV transmission and antiviral resistance in the community [31]. Because mental health services are not integrated within the Indian HIV-care delivery framework [32], linkage to mental health providers falls outside the realm of HIV-programmatic capabilities. Further, such linkages are not always feasible given India’s severe shortage of trained mental health professionals [33]. With mental health conditions on the rise during the current pandemic [34], linkage to care becomes even more challenging. Since anxiety, the deficiency of mental health services and ART nonadherence are interdependent [11], it is not difficult to surmise the negative effect that the pandemic could have on India’s 90–90-90 goals. Notably, we intend to follow up with participants who had GAD-7 scores ≥10 and repeat the assessment after 2 months. In the event of a persistently high GAD-7 score, we will link that individual to a mental health professional at BJGMC-SGH.

**Fig. 1:**
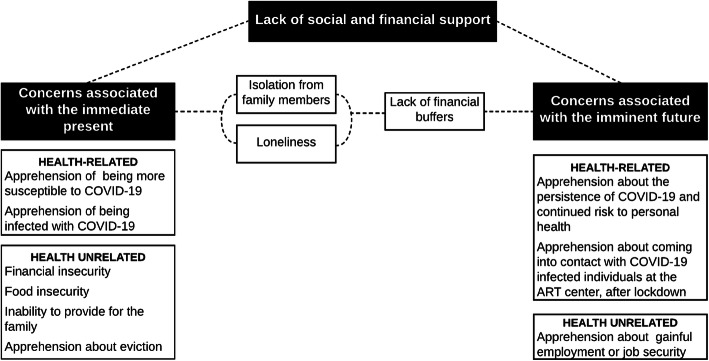
Three themes (black boxes) were identified in thematic analysis as causes of concern. As indicated by dotted lines, the themes were not always mutually exclusive. However, one theme could be a predominant cause of concern. Causes of concern also appeared to recur and their persisience implicated in the absence of mitigating measures. As an example, participants were concerned in the immediate present about not having any money to be able to provide for the family. In the absence of financial buffers such as savings, this concern was also projected into the imminent future

We identified three themes that broadly encapsulate the causes for concern reported across a wide range of GAD-7 scores. As exhibited by participants remarks, one theme could be a predominant cause of concern, but more often than not, themes were interconnected. Thus, a present cause of concern could also be a recurring future concern. It is not untenable to extrapolate from the participants’ statements that the chronicity of a particular concern directly depends on how quickly the concern (financial, social or apprehensions about personal health) is addressed. We have represented this as a conceptual framework in Fig. 1. Although the themes we identified may not seem specific to PLHIV, they must first be contextualized to our participants’ existing socioeconomic backgrounds and the manner in which the pandemic will potentially affect their vertical social mobility. Secondly, the themes need to be understood from the perspective of low socioeconomic status and restricted social mobility and how these will affect HIV treatment outcomes.

More than 50% of our participants earn less than the 2019–2020 estimated monthly per-capita income for India [35], completed less than 10 years of education, and were either employed in the informal sector or were unemployed prior to the lockdown. The Indian government has responded to the financial crisis among the poor with two programs, namely the Pradhan Mantri Garib Kalyan Yojana (PMGKY) and the second tranche of the Atmanirbhar Bharat [36]. These stimulus programs have been criticized as providing a lower level of support than those offered by other governments [36, 37]. Analysis also indicates that instead of mobilizing additional funding, PMGKY has reallocated funding across existing budgets or allowed individuals to make advance withdrawals, raising concerns about the long-term utility of these measures for the poor [36, 38]. Furthermore, the World Bank estimates that the COVID-19 pandemic could push a substantial section of individuals with socioeconomic status similar to our study population into extreme poverty [39]. The association between socioeconomic deprivation and poor mental health is well-established [40]. Worsening mental health is foreseeable for most of our study population and would impact HIV-treatment outcomes (through reduced adherence, increased antiviral resistance, etc.) among a group of disadvantaged individuals within an already vulnerable population.

Interestingly, while none of the participants directly expressed concern about the remaining doses of ART, we found that patients with fewer remaining doses had significantly higher GAD-7 scores compared to those with more doses. This suggests that although concern about ART availability may not be at the forefront, it could be affecting anxiety levels among PLHIV. Notably, a recent survey conducted by the World Health Organization (WHO) showed several countries to be at risk for stock outs of ART [41]. However, the deputy director general of NACO has assured that India will not face such a crisis [42]. This is encouraging news for Asian Indian PLHIV, which will likely go a long way to allaying underlying fears. Indian policy makers need to consider whether access to ART during this pandemic is as uncompromised as the ART stocks.

There are a few limitations to our findings. First, due to the small sample size, our results cannot be extrapolated to all PLHIV in Pune. However, because the socioeconomic backgrounds of patients registered for care at the ART center are largely homogenous, the reported prevalence of anxiety symptoms and consequences of these anxiety levels could be generalizable to them. Second, as we do not have GAD-7 scores prior to the lockdown, we cannot conclude with absolute certainty that the present levels observed are entirely attributable to the pandemic. However, participant concerns were almost exclusively related to the pandemic, the GAD-7 assessed anxiety over the two preceding weeks, and our study was carried out 1 month into the lockdown in India. Therefore, we largely ascribe the observed anxiety levels to the pandemic. It is also difficult to determine whether we have underestimated or overestimated the prevalence of anxiety symptoms, even within our cohort, especially given the high non-response rate. While it may seem likely that patients with higher levels of anxiety would be more apt to refuse our calls, it is equally plausible that responders were more anxious. In addition, the thematic analysis was conducted on responses to a single question, which limits a more nuanced understanding of the issue at hand. However, using thematic analysis as a guiding framework allowed us to more concretely consolidate the wealth of information provided by our participants into definitive themes. Lastly, although we did not observe differences in GAD-7 scores by comorbidity or prior tuberculosis status, we are unable to comment on how mental health in such individuals will change over time given their higher risk for COVID-19 infection [43, 44] and what that will mean in terms of disengagement from care or HIV treatment outcomes for PLHIV with these conditions. We are instituting longitudinal follow-up of all study participants and will better understand these associations by the end of 2020.

## Conclusion

Despite our limitations, our findings provide important insights into the burden and sources of anxiety symptoms in a small group of Asian Indian PLHIV. To our knowledge, these findings are the first to be reported among PLHIV from India during the current pandemic. Our findings also come with the sobering implication that the COVID-19 pandemic will have devastating effects on the mental health of Asian Indian PLHIV as well as downstream HIV-related treatment outcomes, especially as the pandemic continues to grow in India and particularly for PLHIV who are socioeconomically disenfranchised. Sweeping financial assistance along with extensive social and health support mechanisms would indeed be a panacea for COVID-related anxiety symptoms among PLHIV, but are impracticable. Instead, we recommend that HIV care providers regularly use readily available, short screening tools to identify and prioritize PLHIV at risk for anxiety and other mental health conditions. This strategy will not redress the deleterious effects of the pandemic on HIV care but might at least reduce their impact.

## Supplementary information

**Additional file 1.** Crosstabulation between GAD-7 scores and income when stratified by gender. Crosstabulation between GAD-7 scores and living with a spouse when stratified by gender.

## Data Availability

The datasets used and/or analyzed during the current study are available from the corresponding author on reasonable request.

## Abbreviations

PLHIV: People living with HIV
ART: Antiretroviral therapy
BJGMC – SGH: Byramjee jeejeebhoy government medical college and sassoon general hospitals
NACO: National AIDS control organization
GAD-7: General anxiety disorder–7
PMGKY: Pradhan mantri garib kalyan yojana
