# Supplementary material for: The burden of anxiety among people living with HIV during the COVID-19 pandemic in Pune, India
Source: BMC Public Health. 2020 Oct 23;20:1598. doi: 10.1186/s12889-020-09656-8 (PMC7582417; doi:10.1186/s12889-020-09656-8)
Supplement: Supplementary file 1 — Additional file 1. Crosstabulation between GAD-7 scores and income when stratified by gender. Crosstabulation between GAD-7 scores and living with a spouse when stratified by gender. [file 12889_2020_9656_MOESM1_ESM.docx]

**Crosstabulation between GAD-7 scores and income when stratified by gender**

**For cisgender men**

|  | Income <130 USD | Income ≥ 130 USD |
| --- | --- | --- |
| GAD-7<10 | 44.9 % (n =22) | 55.1% (n=27) |
| GAD-7≥10 | 70.6% (n=12) | 29.4% (n=4) |

Fisher’s exact p-value = 0.093

**For cisgender women**

|  | Income <130 USD | Income ≥ 130 USD |
| --- | --- | --- |
| GAD-7<10 | 67.5% (n=52) | 32.5% (n=25) |
| GAD-7≥10 | 52.2% (n=12) | 47.8% (n=11) |

Fisher’s exact p-value = 0.218

**Crosstabulation between GAD-7 scores and living with a spouse when stratified by gender**

**For cisgender men**

|  | Not living with a spouse | Living with a spouse |
| --- | --- | --- |
| GAD-7<10 | 16.3% (n=8) | 83.7% (n=41) |
| GAD-7≥10 | 47.1% (n=8) | 52.9% (n=9) |

Fisher’s exact p-value= 0.020

**For cisgender women**

|  | Not living with a spouse | Living with a spouse |
| --- | --- | --- |
| GAD-7<10 | 70.1% (n=54) | 29.9% (n=23) |
| GAD-7≥10 | 60.9% (n=14) | 39.1% (n=9) |

Fisher’s exact p-value= 0.450
